# Expression of the novel tumour suppressor ***p33^ING1^*** is independent of ***p53***

**DOI:** 10.1054/bjoc.2000.1464

**Published:** 2000-11-22

**Authors:** K-J J Cheung, J A Bush, W Jia, G Li

**Affiliations:** Department of Medicine, Division of Dermatology, Department of Surgery, University of British Columbia and Vancouver Hospital and Sciences Centre, Vancouver, British Columbia, V6H 3Z6, Canada

**Keywords:** *p33^ING1^*, *p53*, in vivo expression, tumour suppressor gene, UV irradiation

## Abstract

A recently cloned tumour suppressor candidate, p33ING1, has been shown in vitro to collaborate with p53 to execute growth arrest and apoptosis. However, it is unclear as to how the expression of ING1 is regulated in normal and stress conditions. Using a p53-knockout mouse model, we investigated if the expression of ING1 was dependent on p53. We found that there was no difference in ING1 mRNA and protein levels between p53+/+ and p53–/– murine organs. In addition, when normal human epithelial keratinocytes (NHEK) and a keratinocyte cell line, HaCaT, which lacks wild-type p53 function, were exposed to UVB irradiation, the expression levels of ING1 were elevated in both NHEK and HaCaT cells. It is interesting, however, that UVB irradiation did not induce ING1 expression in dermal fibroblasts isolated from p53+/+ and p53–/– mice. Based on our findings, we therefore conclude that the expression of ING1 is independent of p53 status. UV induction of ING1 in keratinocytes suggests that ING1 may play a role in cellular stress response and skin carcinogenesis. © 2000 Cancer Research Campaign http://www.bjcancer.com


*British Journal of Cancer* (2000) **83**(11), 1468–1472
© 2000 Cancer Research Campaign
doi: 10.1054/ bjoc.2000.1464, available online at http://www.idealibrary.com on IDEAL®
http://www.bjcancer.com

### Expression of the novel tumour suppressor *p33^ING1^* is independent of *p53*

**K-JJ Cheung Jr[1], JA Bush[1], W Jia[2] and G Li[1]**

[1]Department of Medicine, Division of Dermatology; [2]Department of Surgery, University of British Columbia, and Vancouver Hospital and Sciences Centre, Vancouver, British Columbia, Canada V6H 3Z6

Summary A recently cloned tumour suppressor candidate, p33ING1, has been shown in vitro to collaborate with p53 to execute growth arrest and apoptosis. However, it is unclear as to how the expression of ING1 is regulated in normal and stress conditions. Using a p53-knockout mouse model, we investigated if the expression of ING1 was dependent on p53. We found that there was no difference in ING1 mRNA and protein levels between p53+/+ and p53–/– murine organs. In addition, when normal human epithelial keratinocytes (NHEK) and a keratinocyte cell line, HaCaT, which lacks wild-type p53 function, were exposed to UVB irradiation, the expression levels of ING1 were elevated in both NHEK and HaCaT cells. It is interesting, however, that UVB irradiation did not induce ING1 expression in dermal fibroblasts isolated from p53+/+ and p53–/– mice. Based on our findings, we therefore conclude that the expression of ING1 is independent of p53 status. UV induction of ING1 in keratinocytes suggests that ING1 may play a role in cellular stress response and skin carcinogenesis. © 2000 Cancer Research Campaign http://www.bjcancer.com

**Keywords**: *$p33^{ING1}$*; *p53*; in vivo expression; tumour suppressor gene; UV irradiation

*$p33^{ING1}$*, which encodes a 33-kD nuclear protein, was cloned by subtractive hybridization and subsequent selection of transforming genetic suppressor elements (Garkavtsev et al, 1996). Overexpression of the sense *ING1* inhibits cell growth (Garkavtsev et al, 1996). Specifically, flow cytometry analysis shows that normal fibroblasts transfected with the *ING1* gene are arrested in the $G_0/G_1$ phase of the cell cycle (Garkavtsev et al, 1996). It is observed that this *ING1*-induced arrest is achieved by up-regulating *p53*-dependent activation of the *$p21^{waf1}$* promoter (Garkavtsev et al, 1998). It is further noted that neither *ING1* nor *p53* alone can exert arrest and that both proteins can physically associate with one another, as demonstrated by immunoprecipitation (Garkavtsev et al, 1998). In addition to the function of cell growth arrest, *ING1* has been found to play a role in senescence (Garkavtsev et al, 1997). Expression of *ING1* is 8- to 10-fold higher in senescent cells than in young proliferation-competent human diploid fibroblasts. Cells expressing antisense *ING1* can increase their replicative life by 7-fold. In apoptosis, Helbing and colleagues (1997) reported that elevated expression of *ING1* enhances serum starvation-induced cell death in *p19* mouse teratocarcinoma and NIH 3T3 cells. Recently, another report demonstrates that apoptosis can be enhanced by adenovirus-mediated transfer of both *ING1* and *p53*, further underscoring the interdependent relationship between the two genes (Shinoura et al, 1999). Evidence also suggests that *ING1* is capable of sensitizing cells to chemotherapeutic agents, such as etoposide and γ-irradiation (Garkavtsev et al, 1998). Although the mechanism of such sensitizing ability is unclear, its apoptotic function may be involved.

*p53*, which encodes a nuclear protein, regulates a number of downsteam targets such as *$p21^{waf1}$*, *GADD45*, *bax*, and *bcl-2* and carries out tumour suppressive functions, much like *ING1*. Studies over more than a decade indicate that *p53* is a key mediator of cell cycle regulation, apoptosis, DNA repair, senescence, and sensitization to chemotherapeutic agents (Bond et al, 1994; Miyashita et al, 1994; Smith et al, 1994; Li et al, 1997; 2000; Bunz et al, 1998). Regarded as a 'guardian of the genome', *p53* holds the title of being the most frequently mutated gene known to date (Hollstein et al, 1991). More than 50% of human malignancies of epithelial, mesenchymal, haematopoietic, lymphoid, and central nervous system origin have been shown to contain a mutated form of *p53*. Evidence suggests that loss of normal p53 function is associated with cell transformation in vitro and the development of neoplasms in vivo (Finlay et al, 1989). Under stress conditions, such as those genotoxic in nature, levels of *p53* protein rapidly increase in the cell. The upregulation induces the expression of *$p21^{waf1}$*, a potent inhibitor of cyclin-dependent kinase activity, which inhibits cell cycle progression from $G_0$ to S phase (Shaulsky et al, 1991; El-Deiry et al, 1993). It has also been demonstrated that *p53* maintains genomic stability by enhancing DNA repair and apoptosis. We recently demonstrated that loss of wild-type p53 function results in reduced DNA repair and apoptosis in mouse keratinocytes and fibroblasts after UV irradiation (Li et al, 1996; 1997; 1998*a*; Tron et al, 1998*a*; 1998*b*). As a result of reduced DNA repair and apoptosis, mice with abnormal *p53* function, either by gene knockout or overexpression of mutant *p53*, are predisposed to UV-induced skin cancer development (Li et al, 1995; 1998*b*).

To further our understanding of the relationship between *p53* and *ING1*, we first investigated how the expression of *ING1* behaves in the context of *p53* using a knockout mouse model. We also looked at whether *ING1* expression relies on the status of *p53* in UV-irradiated conditions. Our results indicate that *ING1* expression is independent of wild-type *p53* function and that *ING1* may play a role in cellular stress response and skin carcinogenesis.

*Received 22 March 2000*
*Accepted 18 July 2000*

*Correspondence to:* G Li. E-mail: gangli@interchange.ubc.ca

### MATERIALS AND METHODS

### Animals

*p53*$^{+/+}$ and *p53*$^{-/-}$ mice were purchased from Taconic (New York, NY, USA). *p53*$^{-/-}$ mice carried a disrupted, nonfunctional *p53* gene, created by homologous recombination in an embryonic stem cell line and by microinjection of the stem cells into 3.5-day-old C57BL/6 blastocysts (Donehower et al, 1992).

### Cell culture

Normal human epithelial keratinocytes (NHEK) were obtained from the Tissue Bank of Vancouver General Hospital. They were maintained in Keratinocyte-SFM medium (Canadian Life Technologies, Burlington, Ontario, Canada). Human HaCaT keratinocytes (kindly provided by Dr NE Fusenig, DKFZ, Heidelberg, Germany) were maintained in Dulbecco's Modified Eagle's Medium (DMEM) supplemented with 5% fetal calf serum (Canadian Life Technologies), 100 units ml$^{-1}$ penicillin and 100 µg ml$^{-1}$ streptomycin at 37°C in a 5% $CO_2$ atmosphere.

Dermal fibroblasts of *p53*$^{+/+}$ and *p53*$^{-/-}$ mice were isolated from 4-week-old mice. The mice were sacrificed by cervical dislocation and a 2 × 2 cm skin biopsy was dissected from the dorsal area. The hair was removed and the skin biopsy was disinfected with 2.5% betadine for 1 min, followed by 1 min in 70% ethanol, and washed with phosphate buffered saline (PBS) twice. The skin tissue was then minced and incubated in DMEM containing 200 units ml$^{-1}$ collagenase (Sigma, St Louis, MO, USA) at 37°C for 6 h. The digested skin tissue was centrifuged at 1000 rpm for 10 min and the pellet washed with pre-warmed DMEM twice. The cells were resuspended in DMEM containing 10% FBS and incubated at 37°C in a 5% $CO_2$ atmosphere.

### UVB irradiation

Medium was removed and the cells were exposed to UVB (290–320 nm) using a bank of four unfiltered FS40 sunlamps (Westinghouse, Bloomfield, NJ, USA). The intensity of the UV light was measured by the IL 700 radiometer fitted with a WN 320 filter and an A127 quartz diffuser (International Light, Newburyport, MA, USA). Medium was replaced and cells were incubated in a 5% $CO_2$ incubator at 37°C after UVB irradiation.

### RT-PCR

Total RNA was extracted by TriZol reagent (Canadian Life Technologies) and the concentrations were determined by UV spectrophotometry. 5 µg of total RNA was reverse-transcribed into cDNA in the presence of 10 units µl$^{-1}$ of SUPERSCRIPT II RNase H$^{-}$ Reverse Transcriptase (Canadian Life Technologies), 5X first-strand buffer (250 mM Tris-HCl, pH 8.3, 375 mM KCl, 15 mM $MgCl_2$), 100 mM DTT, 10 mM dNTP mix (10 mM each of dATP, dGTP, dCTP and dTTP at pH 7.0), and 2 pmole of the forward primer (5′-GATCCTGAAGGAGCTAGACG-3′) in a total volume of 20 µl. The RT mix was then incubated at 42°C for 2 min. The reaction was inactivated by heating at 70°C for 15 min. The 100 µl of PCR reaction contained 10% of the first-strand reaction, 10X PCR buffer (200 mM Tris-HCL, pH 8.4, 500 mM KCl), 50 mM $MgCl_2$, 10 mM dNTP Mix, 10 µM of the forward primer (5′-GATCCTGAAGGAGCTAGACG-3′) and 10 mM of the reverse primer (5′-AGAAGTGGAACCACTCGATG-3′), and 5 units µl$^{-1}$ of the *Taq* DNA polymerase. Amplification was carried out as follows: 1. initial denaturation at 94°C for 3 min; 2. denaturation at 95°C for 45 s; 3. annealing at 50°C for 1 min; 4. polymerization at 72°C for 2 min; 5. repeat of steps 2–4 for 40 cycles; and 6. final polymerization at 72°C for 5 min. Samples were then electrophoresed on 1% agarose gels containing 0.5 µg ml$^{-1}$ of ethidium bromide and visualized under UV light. Reaction mix with pCI-*ING1* plasmid DNA (kind gift from Dr K Riabowol, University of Calgary, USA) was used as a positive control. For semi-quantitative PCR, 2 µl of the cDNA samples from reverse transcription were diluted at $10^{-1}$ and $100^{-1}$. They were then amplified by PCR as described above.

### Northern blot analysis

Total RNA was extracted by TriZol reagent and the concentrations were determined by UV spectrophotometry. Samples were heated to 65°C and run on 1% agarose gels containing formaldehyde and 0.5 µg ml$^{-1}$ ethidium bromide. After separation, capillary transfer to nitrocellulose was performed overnight at room temperature and its efficiency assessed by UV light. The blot was then baked for 2 h in a vaccum oven at 80°C. Pre-hybridization was carried out by incubating the blot with a mixture containing 6X SSPE, 5X Denhardt's reagent, 0.5% SDS, and 100 µg ml$^{-1}$ yeast tRNA for 1 h at 65°C. The *ING1* probe was first made by amplifying a 577-bp fragment by PCR using primer 1 (5′-GATCCTGAAG-GAGCTAGACG-3′) and primer 2 (5′-AGAAGTGGAAC-CACTCGATG-3′) and then labelling it with α-$^{32}$ P[dCTP] (10 mCi ml$^{-1}$) according to the manufactured protocol in the Random Primers DNA Labeling System (Canadian Life Technologies). Hybridization was done by incubating the blot with the labelled probe at 65°C for 16–24 h. Filters were washed with 2X SSC/0.1% SDS once for 15 min at room temperature and three washes 20 min each at 65°C. Blots were visualized on X-ray films after an overnight exposure.

### Western blot analysis

Cells were harvested by scraping and solubilized by the Triple detergent lysis buffer containing 50 mM Tris-Cl pH 8.0, 150 mM NaCl, 0.02% sodium azide, 0.1% sodium dodecylsulphate, 1% Nonidet P-40, 100 µg ml$^{-1}$ phenylmethylsulphonyl fluoride, 1 µg ml$^{-1}$ aprotinin, 1 µg ml$^{-1}$ leupeptins and 1 µg ml$^{-1}$ pepstatin A. Concentrations of proteins were determined by the DC Protein Assay (BioRad, Mississauga, Ontario, Canada). 50 µg lane$^{-1}$ of proteins were separated on 10% polyacrylamide/SDS gels and electroblotted onto a nitrocellulose filter. The filter was then blocked with 5% skimmed milk for 1 h and incubated with 1:2000 polyclonal rabbit *p33*$^{ING1}$ antibody (PharMingen, Mississauga, Ontario, Canada) for 1 h at room temperature, followed by three washes with PBS 0.02% Tween-20 (PBS-T). The filter was then incubated with 1:10 000 goat anti-rabbit IgG (Calbiochem, San Diego, CA, USA) at room temperature for 1 h, followed by three washes with PBS-T. The signals were detected with the ECL Western blotting detection system (New England Biolab, Guelph, Ontario, Canada). A 1:2000 dilution of β-actin antibody (Santa Cruz Biotechnology Inc, Santa Cruz, CA, USA) was used as an internal control for each blot.

*© 2000 Cancer Research Campaign*

### Immunohistochemistry

All samples were frozen-sectioned at six-micron and mounted onto saline-coated slides. They were then fixed in cold acetone for 2 min. Using the ImmunoCruz Staining Systems (Santa Cruz Biotechnology Inc) serial sections were first blocked with horse serum for 20 min, then incubated with 1:500 $p33^{ING1}$ polyclonal antibody for 2 h at room temperature, followed by two washes in PBS each for 2 min. Next, the sections were incubated with biotin-labelled anti-rabbit secondary antibody with avidin–biotin–peroxidase complex for 30 min, followed by two washes in PBS each for 2 min and staining with the HRP substrate containing DAB chromogen and peroxidase substrate for 30 s to 5 min. Sections were immediately dehydrated two times in 95% ethanol each for 10 min, two times in 100% ethanol each for 10 s, and three times in xylenes each for 10 s. Slides were added with permanent mounting medium, covered with glass coverslip, and observed under a light microscope.

### RESULTS

### In vivo expression of *ING1* is independent of *p53*

Studies that have examined the relationship between *ING1* and *p53* have mostly been done in vitro (Garkavtsev et al, 1998; Shinoura et al, 1999). Since *p53* is a transcription factor that is known to initiate a whole host of molecular events by transactivating genes, we investigated if *p53* could be an upstream regulator of *ING1* by first examining whether *ING1* was expressed in organs from $p53^{+/+}$ and $p53^{-/-}$ mice. Results from RT-PCR showed that *ING1* was expressed in the brain, liver, lung, heart and skin of both $p53^{+/+}$ and $p53^{-/-}$ mice (Figure 1A). Quantitative RT-PCR indicated that *ING1* mRNA levels were virtually equal in the heart of $p53^{+/+}$ and $p53^{-/-}$ mice (Figure 1B). The data suggest that *ING1* expression is independent of *p53* status. To further confirm the *p53*-independent expression of *ING1*, we used Northern blot analysis to compare mRNA levels in the brain, liver, lung, heart, skin, kidney, testis, and thymus of $p53^{+/+}$ and $p53^{-/-}$ mice. Our results showed that there was no significant difference in *ING1* mRNA expression in all eight organs examined (Figure 1C).

Next, we investigated whether *p53* status affects *ING1* expression at the post-transcriptional level. We compared *ING1* protein levels in the brain, liver, lung, heart, and skin between $p53^{+/+}$ and $p53^{-/-}$ mice. Figure 2 shows that there is no substantial difference in the levels of 33-kD *ING1* protein between $p53^{+/+}$ and $p53^{-/-}$ mice in all five organs examined. Recently, two isoforms of the $p33^{ING1}$ gene, which encode 47-kD and 24-kD proteins, have been found (Saito et al, 2000). The anti-*ING1* antibody we used predominantly detected the 33-kD isoform. To confirm the *p53*-independent expression of *ING1* from the Western analysis, we used immunohistochemical staining to look at *ING1* protein expression in the brain between the $p53^{+/+}$ and $p53^{-/-}$ groups. Our results show that $p53^{+/+}$ and $p53^{-/-}$ mice have similar levels of *ING1* protein (Figure 3).

### UV-induced *ING1* expression is independent of *p53*

*p53* plays a significant role in responding to DNA damage. Stress events, such as ionizing irradiation, ultraviolet light (UV) irradiation and exposure to genotoxic drugs, induce the accumulation of *p53*, which then results in cell cycle arrest, DNA repair and/or apoptosis (El-Diery et al, 1993; Li et al, 1996; 1997; 1998*a*; Tron et al, 1998*b*). Various downsteam *p53*-mediated targets such as $p21^{waf1}$, *GADD45*, *bax*, and *bcl-2* have been shown to be involved in these events. To test the hypothesis that *ING1* may also be a downstream target of *p53* in stress conditions, we exposed normal human epithelial keratinocytes (NHEK) and a keratinocyte cell line (HaCaT), which carries mutated *p53* alleles (Lehman et al, 1993), to UVB irradiation and examined the levels of *ING1* protein. Our results demonstrate that 33-kD *ING1* protein is induced in both NHEK and HaCaT cells after UVB irradiation (Figure 4A and 4B). At 40 mJ cm$^{-2}$, there is a two-fold increase in the level of *ING1* protein in both types of cells. It is also noted that the expression of *ING1* protein in HaCaT cells is dose-dependent, with a maximum induction at 80 mJ cm$^{-2}$. The observation of similar levels of *ING1* induction in NHEK and HaCaT cells by UV irradiation further confirms the *p53*-independent expression of *ING1*.

To confirm that UV induces *ING1* expression at the transcriptional level, *ING1* mRNA levels were examined in HaCaT cells over a time-course. Figure 4C shows that *ING1* mRNA levels increase with time, starting at 4 h and peaking at 24 h.

Using human fibroblasts, Garkavtsev and colleagues (1998) found that DNA damaging agent, adriamycin, did not induce *ING1*

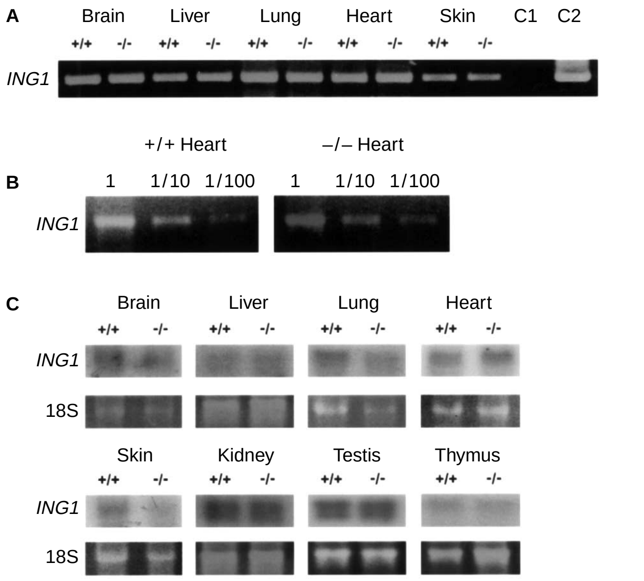

**Figure 1** Analyses of *ING1* mRNA expression of $p53^{+/+}$ and $p53^{-/-}$ organs. (**A**) RT-PCR analysis of mRNA level in different organs of $p53^{+/+}$ and $p53^{-/-}$ mice. C1 = negative control without RNA in the reaction. C2 = positive control with pCI-*ING1* plasmid DNA in the reaction. (**B**) Semi-quantitative RT-PCR analysis of *ING1* mRNA level in the heart of $p53^{+/+}$ and $p53^{-/-}$ mice. A series of dilutions of the *ING1* cDNA was performed and comparison was made between the $p53^{+/+}$ and $p53^{-/-}$ groups. (**C**) Northern blot analysis of *ING1* mRNA expression levels in selected $p53^{+/+}$ and $p53^{-/-}$ organs. The 18S rRNA was used as an internal control

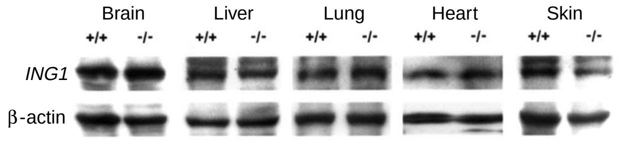

**Figure 2** Western blot analysis of *ING1* expression levels in selected $p53^{+/+}$ and $p53^{-/-}$ organs. β-actin was used as internal control

*© 2000 Cancer Research Campaign*

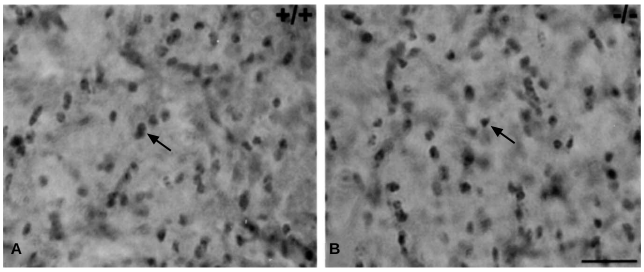

**Figure 3** Immunohistochemical analysis of the brain of $p53^{+/+}$ and $p53^{-/-}$ mice. (**A**) $p53^{+/+}$. (**B**) $p53^{-/-}$. Arrows indicate *ING1* expressing neural cells. Scale bar = 25 μm

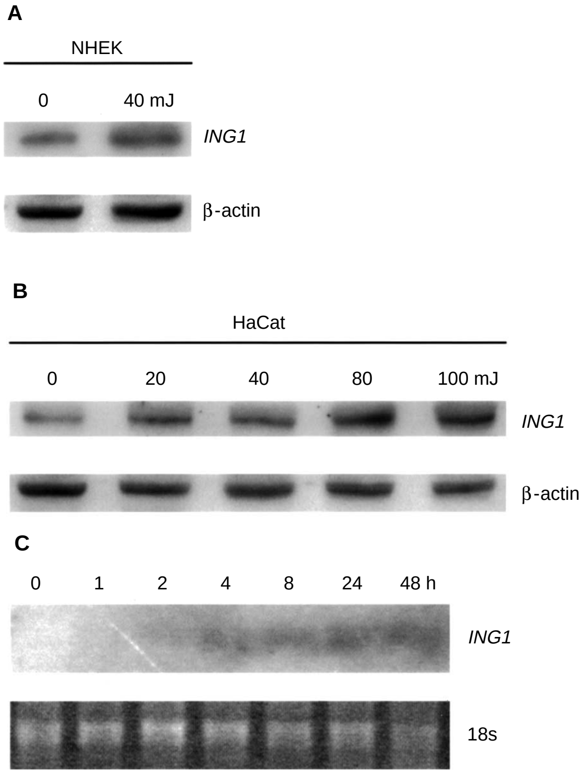

**Figure 4** *ING1* induction by UVB irradiation in keratinocytes. (**A**) and (**B**) Proteins were extracted from the cells 48 h after UVB irradiation and subjected to Western blot analysis. (**A**) NHEK were exposed to UVB at 0 and 40 mJ $cm^{-2}$. (**B**) HaCaT cells were exposed to UVB at 0, 20, 40, 80, and 100 mJ $cm^{-2}$. β-actin was used as an internal control. (**C**) Northern blot analysis of *ING1* expression levels in HaCaT cells. HaCaT cells were exposed to UVB at 80 mJ $cm^{-2}$ and total RNA was collected at 0, 1, 2, 4, 8, 24, and 48 h. The 18S rRNA was used as an internal control

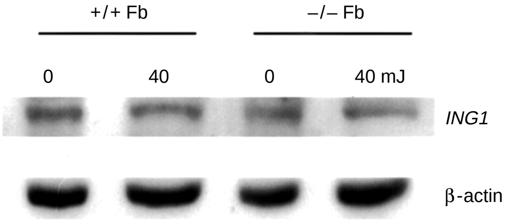

**Figure 5** Western blot analysis of ING1 protein in mouse fibroblasts. $p53^{+/+}$ and $p53^{-/-}$ dermal fibroblasts were exposed to UVB at 0 and 40 mJ $cm^{-2}$. β-actin was used as an internal control expression. To determine if UV-induced *ING1* in keratinocytes was tissue-specific, we exposed dermal fibroblasts isolated from $p53^{+/+}$ and $p53^{-/-}$ mice to 40 mJ $cm^{-2}$ of UVB. Our data show that neither $p53^{+/+}$ nor $p53^{-/-}$ fibroblasts exhibit any change in *ING1* expression (Figure 5). Taken together, we demonstrate that UV-induction of *ING1* is cell type-specific and *p53*-independent.

### DISCUSSION

Our understanding of the biological function of *ING1* has improved over the last 4 years. One of the reasons that this gene has gained increasing attention from the biological community is that, though it has no structural similarity with *p53*, both genes share many of the tumour suppressive functions, including growth arrest, apoptosis, senescence and sensitization to drug treatment. As well, *ING1* has been shown to physically associate with *p53*, further pointing to the importance of its role in carcinogenesis (Garkavtsev et al, 1998). Current studies show that overexpression of *ING1* inhibits cell growth, while chronic expression of *ING1* antisense constructs promotes cell transformation (Garkavtsev et al, 1996; 1998). In addition, it was found that the function of cell growth control is dependent on the activity of both *ING1* and *p53*, and $p21^{waf1}$ has been shown to be their downstream effector (Garkavtsev et al, 1996; 1998).

Although *ING1* shares functional similarities with *p53*, it is not known how the expression of *ING1* is regulated. Since *p53* is a well-known transcriptional factor for many downstream targets (El-Deiry et al, 1993; Miyashita and Reed, 1995; Owen-Schaub et al, 1995), we examined if *p53* had an effect on *ING1* expression in both normal and stress environments. Most studies on *ING1* came primarily from in vitro analysis using long-term cultured cell lines. Since many genetic changes may occur in this type of system, we chose to use the *p53*-knockout mouse model for this study. We compared the *ING1* mRNA and protein levels in a number of organs from $p53^{+/+}$ and $p53^{-/-}$ mice and no significant difference was observed in these two groups (Figures 1–3).

We also investigated if *ING1* could be induced under stress conditions where *p53* is frequently upregulated (Hall et al, 1993; Li et al, 1998*a*). We exposed fibroblasts from $p53^{+/+}$ and $p53^{-/-}$ mice, NHEK and a keratinocyte cell line, HaCaT, to UVB and compared the *ING1* protein levels in these cells. HaCaT is a spontaneously immortalized, non-tumorigenic human keratinocyte cell line that behaves phenotypically like its normal counterpart in terms of patterns of growth and differentiation (Boukamp et al, 1988). Besides the advantage of being similar in many respects with its normal counterpart, this cell line lacks the functional *p53* gene, allowing the study of the relationship between *p53* and its associates. Our results indicate that *ING1* is induced in both NHEK and HaCaT cells, regardless of *p53* status, further confirming our previous observation of *ING1 p53*-independent expression. The fact that *ING1* is not induced in fibroblasts after UV irradiation suggests that UV-induction of *ING1* expression is cell type-specific, consistent with the data reported by Garkavtsev and colleagues (1998) where they found no induction of *ING1* expression in fibroblasts treated with adriamycin, a chemotherapeutic drug. Similarly, Zeremski et al (1999) reported that there was a lack of effect of *p53* status on *ING1* levels in murine mammary gland cell lines under normal and stress conditions.

Keratinocyte-specific UVB induction of *ING1* may have biological significance, as keratinocytes are the primary target of

© 2000 Cancer Research Campaign

UVB in the skin. The induced *ING1* protein may contribute to the various cellular responses, such as apoptosis and DNA repair, after UVB irradiation.

In this study, we found that wild-type *p53* is not required for the expression of *ING1* in both normal and stress conditions, although their partnership has been shown to be required for exerting biological effects such as growth inhibition and apoptosis (Garkavtsev et al, 1998; Shinoura et al, 1999). The UV induction of *ING1* seen in our keratinocytes therefore suggests two possibilities: 1. *ING1* is capable of responding to stress in terms of expression independent of *p53* but cannot perform its cellular activities without the functional *p53*; and 2. *ING1* is capable of responding to stress and carries out its functions independent of *p53*. Further functional studies are needed to decipher the role of *ING1* in cellular stress response. Because we have previously shown that *p53* gene knockout predisposes animals to UV-induced skin cancer development, reduces UV-damaged DNA repair, and lowers the apoptotic rate (Li et al, 1995; 1996; 1997; 1998*a*; Tron et al, 1998*b*), investigations on how *ING1* cooperates with *p53* in keratinocytes to modulate cell cycle arrest, DNA repair, and apoptosis will further determine the functional significance of *ING1* in UV-induced stress response in particular and skin carcinogenesis in general. To support the notion that *ING1* plays a role in skin carcinogenesis, we have recently found that UV-induced murine squamous carcinomas express significantly higher levels of *ING1* regardless of *p53* status in comparison to normal skin (data not shown). Although current mutational studies indicate that very few alterations are found in *ING1* gene in breast, ovarian, and gastric cancers (Ohmori et al, 1999; Oki et al, 1999; Toyama et al, 1999), *ING1* mutation may only be specific to UV-induced skin cancers. Future studies on *ING1* mutational status in skin tumours will help reveal the nature of its *ING1* induction.

Taken together, our results demonstrate that *ING1* expression is independent of *p53* status and that UV-induction of *ING1* in human keratinocytes suggests a role of *ING1* in cellular stress response and skin carcinogenesis.

### ACKNOWLEDGEMENTS

This project is supported by the Canadian Dermatology Foundation and the Medical Research Council of Canada. Dr Gang Li is a recipient of Young Scientist Award from Vancouver Hospital and Health Sciences Centre.

### REFERENCES

Bond JA, Wyllie FS and Wynford-Thomas D (1994) Escape from senescence in human diploid fibroblasts induced directly by mutant p53. *Oncogene* **9**: 1885–1889

Boukamp P, Petrussevska RT, Breitkreutz D, Hornung J, Markham A and Fusenig NE (1988) Normal keratinization in a spontaneously immortalized aneuploid human keratinocyte cell line. *J Cell Biol* **106**: 761–771

Bunz F, Dutriaux A, Lengauer C, Waldman T, Zhou S, Brown JP, Sedivy JM, Kinzler KW and Vogelstein B (1998) Requirement for p53 and p21 to sustain G2 arrest after DNA damage. *Science* **282**: 1497–1501

Donehower LA, Harvey M, Slagle BL, McArthur MJ, Montgomery CA Jr, Butel JS and Bradley A (1992) Mice deficient for p53 are developmentally normal but susceptible to spontaneous tumours. *Nature* **356**: 215–221

El-Deiry WS, Tikino T, Velculescu VE, Levy DB, Parsons R, Trent JM, Lin D, Mercer WE, Kinzler KW and Vogelstein B (1993) WAF1, a potential mediator of p53 tumour suppression. *Cell* **75**: 805–816

Finlay CA, Hinds PW and Levine AJ (1989) The p53 proto-oncogene can act as a suppressor of transformation. *Cell* **57**: 1083–1093

Garkavtsev I, Kazarov A, Gudkov A and Riabowol K (1996) Suppression of the novel growth inhibitor p33ING1 promotes neoplastic transformation. *Nat Genet* **14**: 415–420

Garkavtsev I and Riabowol K (1997) Extension of the replicative life span of human diploid fibroblasts by inhibition of the p33ING1 candidate tumour suppressor. *Mol Cell Biol* **17**: 2014–2019

Garkavtsev I, Grigorian IA, Ossovskaya VS, Chernov MV, Chumakov PM and Gudkov AV (1998) The candidate tumour suppressor p33ING1 cooperates with p53 in cell growth control. *Nature* **391**: 295–298

Hall PA, McKee PH, Menage HD, Dover R and Lane DP (1993) High levels of p53 protein in UV-irradiated normal human skin. *Oncogene* **8**: 203–207

Helbing CC, Veillette C, Riabowol K, Johnston RN and Garkavtsev I (1997) A novel candidate tumour suppressor, ING1, is involved in the regulation of apoptosis. *Cancer Res* **57**: 1255–1258

Hollstein M, Sidransky D, Vogelstein B and Harris CC (1991) p53 mutations in human cancers. *Science* **253**: 49–53

Lehman TA, Modali R, Boukamp O, Stanek J, Bennett WP, Welsh JA, Metcalf RA, Stampfer MR, Fusenig N and Rogan EM (1993) p53 mutations in human immortalized epithelial cell lines. *Carcinogenesis* **14**: 833–839

Li G, Ho VC, Berean K and Tron VA (1995) Ultraviolet radiation induction of squamous cell carcinomas in p53 transgenic mice. *Cancer Res* **55**: 2070–2074

Li G, Mitchell DL, Ho VC, Reed JC and Tron VA (1996) Decreased DNA repair but normal apoptosis in ultraviolet-irradiated skin of p53-transgenic mice. *Am J Pathol* **148**: 1113–1123

Li G, Ho VC, Mitchell DL, Trotter MJ and Tron VA (1997) Differentiation-dependent p53 regulation of nucleotide excision repair in keratinocytes. *Am J Pathol* **150**: 1457–1464

Li G and Ho VC (1998*a*) p53-dependent DNA repair and apoptosis respond differently to high- and low-dose ultraviolet radiation. *Br J Dermatol* **139**: 3–10

Li G, Tron V and Ho V (1998*b*) Induction of squamous cell carcinoma in p53-deficient mice after ultraviolet irradiation. *J Invest Dermatol* **110**: 72–75

Li G, Bush JA and Ho VC (2000) p53-dependent apoptosis in melanoma cells after treatment with camptothecin. *J Invest Dermatol* **114**: 514–519

Miyashita T and Reed JC (1995) Tumour suppressor p53 is a direct transcriptional activator of the human bax gene. *Cell* **80**: 293–299

Miyashita T, Krajewski S, Krajewska M, Wang HG, Lin HK, Liebermann DA, Hoffman B and Reed JC (1994) Tumour suppressor p53 is a regulator of bcl-2 and bax gene expression in vitro and in vivo. *Oncogene* **9**: 1799–1805

Ohmori M, Nagai M, Tasaka T, Koeffler HP, Riabowol K and Takahara J (1999) Decreased expression of p33ING1 mRNA in lymphoid malignancies. *Am J Hematol* **62**: 118–119

Oki E, Maehara Y, Tokunaga E, Kakeji Y and Sugimachi K (1999) Reduced expression of p33(ING1) and the relationship with p53 expression in human gastric cancer. *Cancer Lett* **147**: 157–162

Owen-Schaub LB, Zhang W, Cusack JC, Angelo LS, Santee SM, Fujiwara T, Roth JA, Deisseroth AB, Zhang WW, Kruzel E and Radinsky R (1995) Wild-type human p53 and a temperature-sensitive mutant induce Fas/APO-1 expression. *Mol Cell Biol* **15**: 3032–3040

Saito A, Furukawa T, Fukushige S, Koyama S, Hoshi M, Hayashi Y and Horii A (2000) p24/ING1-ALT1 and p47/ING1-ALT2, distinct alternative transcripts of p33/ING1. *J Hum Genet* **45**: 177–181

Shaulsky G, Goldfinger N, Tosky MS, Levine AJ and Rotter V (1991) Nuclear localization is essential for the activity of p53 protein. *Oncogene* **6**: 2055–2065

Shinoura N, Muramatsu Y, Nishimura M, Yoshida Y, Saito A, Yokoyama T, Furukawa T, Horii A, Hashimoto M, Asai A, Kirino T and Hamada H (1999) Adenovirus-mediated transfer of p33ING1 with p53 drastically augments apoptosis in gliomas. *Cancer Res* **59**: 5521–5528

Smith ML, Chen IT, Zhan Q, Bae I, Chen CY, Gilmer TM, Kastan MB, O'Connor PM and Fornace AJ Jr (1994) Interaction of the p53-regulated protein Gadd45 with proliferating cell nuclear antigen. *Science* **266**: 1376–1380

Toyama T, Iwase H, Watson P, Muzik H, Saettler E, Magliocco A, DiFrancesco L, Forsyth P, Garkavtsev I, Kobayashi S and Riabowol K (1999) Suppression of ING1 expression in sporadic breast cancer. *Oncogene* **18**: 5187–5193

Tron VA, Trotter MJ, Ishikawa T, Ho VC and Li G (1998a) p53-dependent regulation of nucleotide excision repair in murine epidermis in vivo. *J Cutan Med Surg* **3**: 16–20

Tron VA, Trotter MJ, Tang L, Krajewska M, Reed JC, Ho VC and Li G (1998b) p53-regulated apoptosis is differentiation dependent in ultraviolet B-irradiated mouse keratinocytes. *Am J Pathol* **153**: 579–585

Zeremski M, Hill J, Kwek S, Grigorian I, Gurova K, Garkavtsev I, Diatchenko L, Koonin E and Gudkov A (1999) Structure and regulation of the mouse ing1 gene. *J Biol Chem* **274**: 32172–32181

 *© 2000 Cancer Research Campaign*
